# Heterogeneity in endocrine monitoring and timing of endocrinology involvement during immune checkpoint inhibitor therapy: A Real-World Single-Center study

**DOI:** 10.1007/s12020-026-04589-5

**Published:** 2026-04-01

**Authors:** Selin Tekin, Seda Hanife Oguz, Süleyman Nahit Sendur, Ugur Ünlütürk, Tomris Erbas, Selcuk Dagdelen

**Affiliations:** Faculty of Medicine, Department of Endocrinology & Metabolism, Hacettepe University, Ankara, Türkiye

**Keywords:** Immune checkpoint inhibitors (ICIs), Endocrine immune-related adverse events (irAEs), Endocrinology

## Abstract

**Purpose:**

Endocrine immune-related adverse events (irAEs) are increasingly encountered with immune checkpoint inhibitors (ICIs), yet patterns of screening, referral, and emergency outcomes remain underexplored. This study evaluated the adequacy of endocrine monitoring, iming of endocrinology involvement, and determinants of endocrine emergencies in ICI-treated patients.

**Methods:**

We retrospectively reviewed 103 patients (67 men, 36 women; mean age 60.8 ± 12.1 years) referred to the Endocrinology Department between 2016 and 2025. Clinical data, cancer types, immunotherapy regimens, hormonal assessments, and causes and timing of referral were analyzed.

**Results:**

Most patients (93.3%) were referred due to newly developed endocrine irAEs, with endocrinology involvement occurring at a median of 7.4 months (≈ 10th therapy cycle) after ICI initiation. The predominant ICI regimen involved PD-1 inhibitors (66%). Thyroid dysfunction (*n* = 64), secondary adrenal insufficiency (*n* = 28), and hyperglycemia (*n* = 4) were the leading endocrinopathies; hypophysitis was detected in 7 cases. Before ICI treatment, endocrine evaluation was absent in 22.3% and incomplete in 38.2%, while 9.7% received no endocrine follow-up during ICI therapy. Emergency admissions occurred in 17.4%, mainly due to adrenal crisis (*n* = 12), followed by thyrotoxicosis (*n* = 3). Although baseline screening status and timing of endocrinology involvement were not associated with emergency risk, phenotype strongly influenced outcomes: central adrenal insufficiency increased risk (RR = 3.29; 95% CI 1.33–8.08; *p* = 0.0197).

**Conclusions:**

Endocrine irAEs are frequent and may be recognized only after clinical deterioration, exposing patients to preventable endocrine emergencies, particularly adrenal crisis. These findings highlight heterogeneity and gaps in endocrine surveillance during ICI therapy and support early, structured oncologist–endocrinologist collaboration with standardized baseline and follow-up assessments to improve patient safety and continuity of immunotherapy.

## Introduction

Immunotherapies are revolutionary new generation agents in cancer treatment. These medications serve as immune check point inhibitors (ICIs) that facilitate T-cells to recognize and attack cancer cells Bagchi, Yuan [1]. As the use of these agents in oncology practice continues to expand, immune-related adverse events (irAEs) have emerged as significant challenges for both clinicians and patients [2, 3].

Endocrine irAEs are among the most common irAEs and include a range of conditions, including thyroid dysfunction, adrenal insufficiency (primary or secondary), hypophysitis, immune-mediated diabetes mellitus, and, more rarely, parathyroid dysfunction [2, 4, 5]. In the majority of cases, endocrinopathies emerge within the initial weeks or months of ICI therapy [6], however, they can also occur at any point during treatment, including after discontinuation of therapy [7]. The incidence and clinical profile of endocrine irAEs varies based on immunotherapy type and treatment approach. For instance, thyroid dysfunction is more commonly associated with programmed death protein 1 (PD-1) and programmed death protein ligand 1 (PD-L1) inhibitors, with hypothyroidism rates reaching up to 40% in patients receiving anti-PD-1 therapy [8, 9]. On the other hand, hypophysitis is more frequently linked to cytotoxic T-lymphocyte–associated antigen 4 (CTLA-4) inhibitors [10]. Additionally, endocrine irAEs follow distinct clinical courses depending on the specific immunotherapy used; for example, anti-PD-1-associated hypophysitis primarily manifests as isolated adrenocorticotropic hormone (ACTH) deficiency, whereas anti-CTLA-4 therapy more commonly causes panhypophysitis [11, 12]. Although less frequent than thyroid or pituitary dysfunction, pancreatic irAEs can be life-threatening, with PD-1 and PD-L1 inhibitors associated with a 1% incidence of immune-mediated diabetes mellitus, often presenting as new-onset Type 1 diabetes or worsening of preexisting Type 2 diabetes [13, 14].

Despite the well-documented endocrine complications of ICIs and the availability of guidelines from major societies such as American Society of Clinical Oncology (ASCO) European Society for Medical Oncology (ESMO), and the Endocrine Society [15–17] real-world implementation remains inconsistent. Patients on immunotherapy continue to receive inadequate endocrine surveillance, and referrals to endocrinologists are often delayed or overlooked, leading to preventable complications and emergency presentations [14, 15]. One possible explanation is that distinguishing the symptoms and signs of endocrinopathies from those of the cancer itself or other treatments administered alongside ICI can pose a challenge [18, 19]. However, it is important to note that, while most endocrine irAEs being graded as mild, they can cause permanent damage or life-threatening crises, necessitating lifelong replacement therapy and potentially requiring immunotherapy cessation [2]. Tailored endocrine monitoring and early recognition are therefore essential to optimize patient management.

This study aims to evaluate the referral patterns, diagnostic approaches, and time to endocrinology consultations for patients receiving ICIs. By identifying gaps in real-world practice, we seek to underscore the need for endocrinologists to be more actively involved in managing irAEs and advocate for better interdisciplinary collaboration between oncology and endocrinology to improve patient outcomes.

## Materials and methods

This single-center cohort study was conducted between January 2016 and June 2025 at the Hacettepe University School of Medicine, Department of Endocrinology and Metabolism. A total of 103 medical records of patients referred from the medical oncology departmen**t** to the endocrinology department, either during or prior to receiving immunotherapy, were reviewed retrospectively. The study was approved by the Hacettepe University Ethics Committee (Approval No: SBA 25/545).

Demographic characteristics of the patients, including age and gender, were recorded. The type of oncological malignancy and the immunotherapy regimen used in treatment were documented, categorized as either single-agent therapy or combination therapy. Immunotherapy agents were further classified based on the most recent classification system, including PD-1 inhibitors, PD-L1 inhibitors, and CTLA-4 inhibitors [15]. The timing of referrals were categorized as pre-treatment assessment (before to starting ICIs) and ongoing assessment (while receiving ICIs). The reasons for referrals were categorized as evaluation for endocrine dysfunctions related or unrelated to ICIs. Furthermore, the impact of delays in endocrinology consultations was evaluated, with a focus on urgent endocrine-related complications that required emergency medical intervention. Additionally, we examined whether endocrinological evaluations were conducted before and during immunotherapy, as well as the methods used to assess endocrine axis, including:

• **Hypothalamic-Pituitary-Thyroid (HPT) Axis** (thyroid-stimulating hormone [TSH], free thyroxine [fT4], free triiodothyronine [fT3])

• **Hypothalamic–Pituitary–Adrenal (HPA) Axis** (adrenocorticotropic hormone [ACTH] and serum cortisol levels)

• **Hypothalamic–Pituitary–Gonadal (HPG) Axis** (follicle-stimulating hormone [FSH], luteinizing hormone [LH], estradiol in females, and total testosterone in males)

• **Growth Hormone–Insulin Like Growth Factor-1 (GH–IGF-1) Axis** (growth hormone [GH] and insulin-like growth factor-1 [IGF-1] levels)

• **Prolactin (PRL) levels**.

• **Parathyroid Hormone (PTH) levels**, serum calcium and phosphate levels.

• **Glycemic assessment** (fasting plasma glucose and glycated hemoglobin [HbA1c] levels, C-peptide)

The sufficiency and appropriateness of these assessments were classified as follows:

- **Complete**: The axis was evaluated with all relevant parameters.
- **Partial**: The axis was assessed but not with all required parameters.
- **Not Performed**: The axis was not assessed.

Endocrine irAEs were defined in accordance with the European Society of Endocrinology Clinical Guideline on ICI-induced endocrinopathies [15]. The diagnostic criteria for each subtype were as follows;

**Pituitary irAEs**.

- **Isolated ACTH Deficiency**:

Defined as a low morning serum cortisol level (< 3 µg/dL) accompanied by a low or inappropriately normal ACTH concentration, in the absence of other pituitary hormone deficiencies, after excluding exogenous glucocorticoid exposure (e.g., dexamethasone or other systemic glucocorticoid therapy) and other causes of secondary adrenal suppression.

- **Multiple Pituitary Hormone Deficiencies**:

Defined as the presence of two or more anterior pituitary hormone deficiencies, with or without radiological evidence of pituitary involvement.

- **Hypophysitis**:

Hypophysitis was diagnosed when multiple anterior pituitary hormone deficiencies were accompanied by characteristic MRI findings such as diffuse pituitary enlargement, homogeneous contrast enhancement, or stalk thickening.

- **Thyroid irAE**

Defined based on serum thyroid function tests. Thyrotoxicosis was diagnosed as suppressed TSH with elevated free T4 and/or free T3 levels. Hypothyroidism was defined as elevated TSH with reduced free T4 levels. A biphasic course transitioning from thyrotoxicosis to hypothyroidism was suggestive of ICI-induced destructive thyroiditis. Thryoid autoantibodies including anti-thyroid peroxidase (Anti-TPO Ab), anti-thyroglobulin (Anti-Tg Ab), and thyroid stimulating immunoglobulin (TSI) were all checked as indicated.

- **ICI-Related Diabetes Mellitus (irDM)**

Defined as new-onset insulin-dependent diabetes following ICI treatment, characterized by hyperglycemia (FPG ≥ 126 mg/dL or random glucose ≥ 200 mg/dL), low or absent C-peptide, and/or diabetic ketoacidosis.The presence of diabetes-related autoantibodies was also evaluated (GAD65, IA-2, IAA, ZnT8, and ICA). Preexisting diabetes or other etiologies were excluded.

- **Hypoparathyroidism**

Defined by the presence of hypocalcemia (corrected serum calcium < 8.5 mg/dL or ionized calcium < 1.12 mmol/L) with simultaneously low or inappropriately normal intact PTH levels, in the absence of alternative causes such as surgical or radiation-induced hypoparathyroidism.

## Statistical analysis

All statistical analyses were performed using IBM SPSS Statistics for Windows, version 25.0 (IBM Corp., Armonk, NY, USA**).** Descriptive statistics were used to summarize the study population’s baseline characteristics. Continuous variables were tested for normality using the Kolmogorov-Smirnov test and presented as mean ± standard deviation (SD) for normally distributed data or median (interquartile range, IQR) for non-normally distributed data. Categorical variables were reported as frequencies and percentages (%).

For univariate modelling, we fitted single-predictor logistic regression models. For each predictor we reported the coefficient (B, log-odds), standard error (SE), Wald chi-square (Wald χ²**)**, two-sided p-value (p), and the odds ratio (OR) with 95% confidence interval (CI). For binary 2 × 2 comparisons we additionally reported Fisher’s exact p-values and crude risk ratios (RR**)** with 95% CIs using the Katz method; when a cell count was zero, a 0.5 continuity correction was applied for CI stability. All tests were two-sided, and results with *p* < 0.05 were considered statistically significant.

## Results

In this study, 103 patients (67 males / 36 females) with a mean age of 60.8 ± 12.1 years, who were referred to our department were assessed. The most common indication for immunotherapy was lung cancer (32.1%), followed by renal cell carcinoma (15.5%) and gynecologic cancers (9.7%), Table 1. The majority of patients were receiving single-agent immunotherapy (78.2%), with PD-1 inhibitors being the most common (*n* = 68), followed by PD-L1 inhibitors (*n* = 12), and CTLA-4 inhibitors (*n* = 1). The remaining 21.8% were on combination regimens.

**Table 1:** Characteristics of patients referred to endocrinology due to Immunotherapy-Related endocrine dysfunction

Variable	
**Total Patients Referred to Endocrinology (n)**	103
**Age (mean ± SD**,** years)**	60.6 ± 12.5
**Gender (n)**	
Female Male	36 (35.3) 67 (64.7)
**Timing of Referral (median)**	
Months after ICI initiation Therapy cycle	7.4
	10th
**Oncologic Malignancies n (%)**	
Lung cancer	33 (32.1)
Renal cell cancer	16 (15.5)
Gynecologic cancers	10 (9.7)
Malignant melanoma	9 (8.7)
GIS cancers	9 (8.7)
Breast cancer	6 (5.8)
Bladder cancer	5 (4.9)
Larynx cancer	4 (3.9)
Hepatocelluler cancer	3 (2.9)
Lymphoma	3 (2.9)
Sarcomas	3 (2.9)
Others	2 (1.9)
**Reason of Referral n (%)**	
Pre-treatment Assessment	2 (1.9)
Ongoing Assessment Without Endocrine Dysfunction	3 (2.9)
Other Endocrine Abnormalities (e.g., thyroid nodule evaluation, preoperative endocrine assessment, etc.)	2 (1.9)
Endocrine Dysfunction	96 (93.3)
*Thyroid test abnormalities (n)*	64
*Hypocortisolism (n)*	28
*Hyperglycemia (n)*	4
**Emergency admissions due to endocrine dysfunction n (%)**	18 (17.4)
Hypocortisolism (n)	12
Thyrotoxicosis (n)	3
Hypothyroidism (n)	1
Hypergylcemia (n)	2

ICI: Immune Checkpoint Inhibitor

The majority of patients (93.3%) were referred to the endocrinology department due to the development of endocrine irAEs, whereas referrals prior to treatment initiation (1.9%) and for assessment before the onset of endocrinopathies during ongoing therapy (2.9%) were rare. The most frequent endocrine dysfunction leading to referral was thyroid test abnormalities (*n* = 64), followed by hypocortisolism (*n* = 28) and hyperglycemia (*n* = 4). Endocrinology consultations were typically requested, on average, 7.4 months after the initiation of immunotherapy, most commonly following the completion of the 10th cycle of immunotherapy, as shown in Table 1.

Following endocrinological evaluation, 94.1% of patients (*n* = 97) were diagnosed with an endocrine dysfunction, with thyroiditis being the most common endocrinopathy **(***n* = 65). The majority of patients presented with hyperthyroidism (*n* = 50), followed by secondary adrenal insufficiency (*n* = 28), which most frequently manifested as isolated ACTH deficiency (*n* = 24), while a minority had multiple hormone deficiencies (*n* = 4). Among the 28 patients with hypocortisolism, pituitary magnetic resonance imaging (MRI) revealed hypophysitis in 7 cases. Hyperglycemia was less frequent (*n* = 4). As expected, thyroiditis and isolated ACTH deficiency were both predominantly associated with PD-1 inhibitors, Table 2.

**Table 2:** Immunotherapy Classes and Related Endocrinopathies

Treatment Regimen	Endocrine irAEs (*n*)		
	Thyroiditis	Adrenal Insufficiency	Diabetes Mellitus
**PD-1 inhibitors**	45	19 (5*)	0
**PD-L1 inhibitors**	8	2	3
**CTLA-4 inhibitors**	1	0	0
**Combination Regimen**	11	7 (2*)	1
**Total**	65	28 (7*)	4

PD-1: programmed cell death protein 1, PD-L1: programmed death-ligand 1, CTLA-4: Cytotoxic T-lymphocyte-associated protein 4, (*) Patients with pituitary MRI findings compatible with hypophysitis

**Fig. 1:**
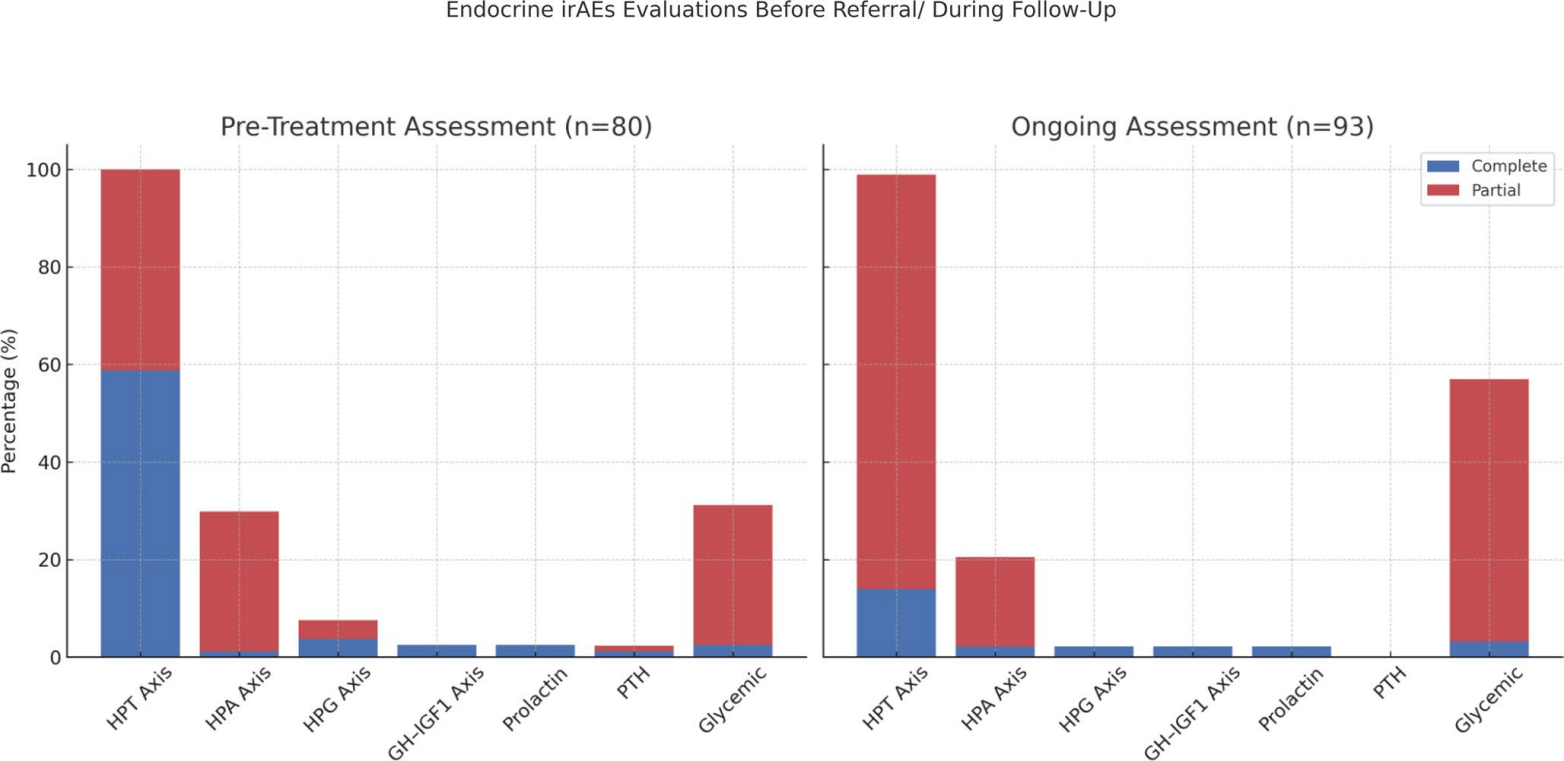
Endocrine irAEs Evaluation Before Referral/During Follow-up

An examination on oncology follow-up records showed that 22.3% of patients had not undergone any endocrinological biochemical evaluations prior to immunotherapy. The most frequently screened endocrine axis before ICI initiation was the HPT axis (*n* = 80), while only one patient underwent a complete endocrinological evaluation. Additionally, 9.7% of patients did not undergo any endocrine evaluations during the course of immunotherapy. Among patients who underwent endocrinological evaluation, the thyroid axis was the most commonly assessed (100%), followed by HPA axis (25%), HPG axis (2.4%), prolactin levels (2.4%), and GH-IGF-1 axis (2.4%). PTH levels were not evaluated in any patients (0%), and none developed hypocalcemia throughout the course of ICI therapy Glycemic assessment (fasting plasma glucose and/or HbA1c) was performed in 51.9% of patients. Thyroid axis evaluation was primarily conducted using TSH and fT4 levels. Screening of the adrenal axis was predominantly conducted using morning cortisol measurements, while complete adrenal axis evaluation was performed in only 2 patients. Notably, no patient underwent a comprehensive endocrinological assessment, as shown in Fig. 1.

**Fig. 2:**
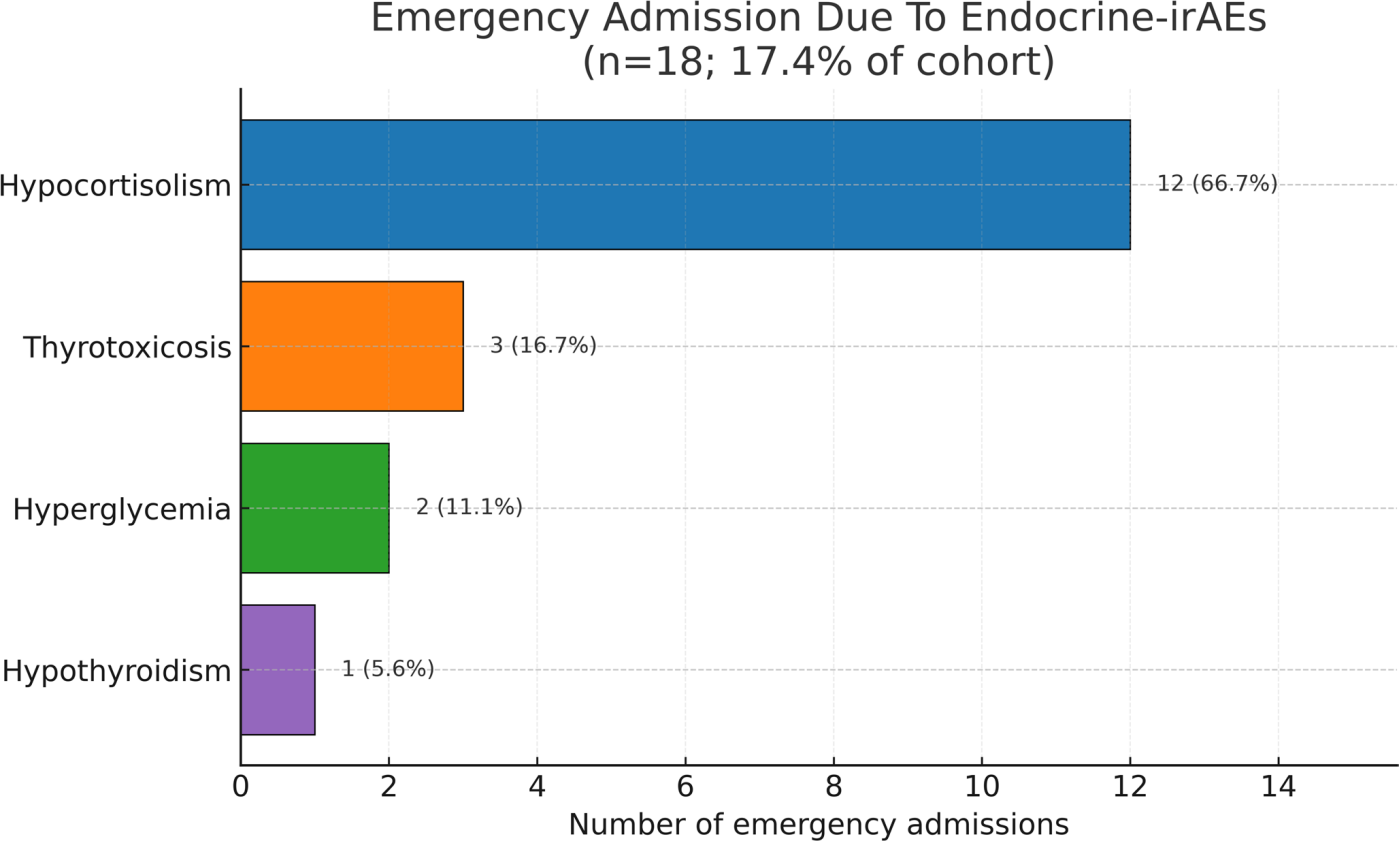
Emergency Admission Due-to Endocrine irAEs

Urgent endocrinopathy related admissions occurred in 17.4% of patients prior to endocrinology consultation, primarily due to hypocortisolism (*n* = 12), followed by thyrotoxicosis (*n* = 3), hyperglycemia (*n* = 2) and hypothyroidism (*n* = 1), as shown in Fig. 2. Most of the patients with acute adrenal insufficiency admitted to the emergency department with fatigue and exhaustion, and low plasma cortisol levels were detected. High-dose glucocorticoid therapy was initiated accordingly. Patients with thyrotoxicosis presented with tachycardia and rhythm abnormalities, whereas hypothyroidism manifested as dizziness and fatigue. None of the patients experienced mortality due to these conditions.

Univariate logistic regression predicting emergency admission found no significant predictors; referral time (OR 0.98, 95% CI 0.93–1.03; *p* = 0.490), baseline endocrine screening (OR 1.74, 0.63–4.84; *p* = 0.288), or age (per year: OR 1.01, 0.96–1.05; *p* = 0.808), Table 3.

**Table 3:** Univariate Logistic Regression for Emergency Admission

Predictor	B	SE	Wald (χ²)	*p*	OR (95% CI)
**Referral Time (month)**	-0.019	0.027	0.495	0.490	0.981 (0.93–1.03)
**Baseline Endocrine Screening (yes/no)**	0.555	0.522	1.130	0.288	1.742 (0.63–4.85)
**Age at Referral (years)**	0.005	0.021	0.057	0.808	1.005 (0.96–1.05)

coefficient (B, log-odds), standard error (SE), Wald chi-square (Wald χ²), two-sided p-value (p), and the odds ratio (OR) with 95% confidence interval (CI)

**Fig. 3:**
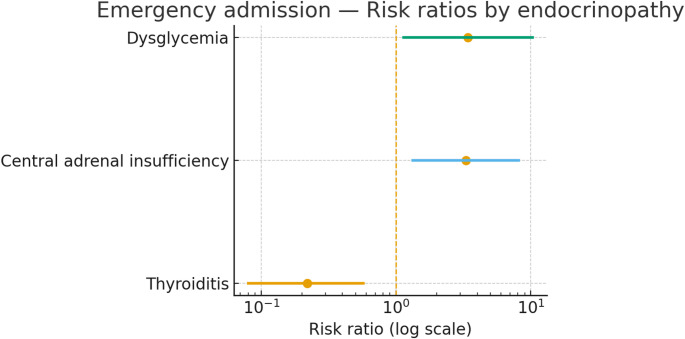
Emergency Admission- Risk Ratios by Endocrinopathy

Among endocrinopathy phenotypes, risk of emergency admission differed substantially. Thyroiditis was associated with a lower risk (crude RR = 0.22; 95% CI, 0.08–0.58; Fisher *p* = 0.0016). In contrast, central adrenal insufficiency showed a higher risk (crude RR = 3.29; 95% CI, 1.33–8.08; Fisher *p* = 0.0197). Dysglycemia showed an elevated point estimate but was not statistically significant (crude RR = 3.42; 95% CI, 1.14–10.29; Fisher *p* = 0.121), reflecting small cell counts, Fig. 3.

## Discussion

This study highlights heterogeneity and real-world gaps in endocrine monitoring during ICI therapy. Endocrinology involvement occurred at a median of 7.4 months after ICI initiation and is best interpreted as time-to-specialist involvement rather than a direct measure of referral delay. Monitoring practices differed substantially across endocrine axes. Thyroid function tests were the most consistently assessed in routine oncology follow-up, whereas adrenal surveillance was less systematic and often symptom-driven, which may delay recognition of central adrenal insufficiency. Although random glucose values may be available as part of routine chemistry panels prior to ICI infusions, structured glycemic surveillance (e.g., fasting glucose and/or HbA1c with trend evaluation) was not uniformly performed or consistently documented in our cohort. Taken together, these findings highlight actionable gaps in routine care and support the need for more consistent and structured endocrine monitoring strategies during ICI therapy.

Endocrine irAEs are increasingly recognized in both endocrinology and oncology literature, with growing awareness among healthcare professionals [20–22]. However, despite significant advancements in understanding these adverse events, real-world data on the implementation of guideline recommendations and the adequacy of endocrine evaluation during immunotherapy remain limited [14]. Our single-center experience highlights that endocrine-related toxicities are frequently overlooked in patients receiving immune checkpoint inhibitors, with endocrine assessments during oncology follow-ups often being insufficient or incompatible. The application of these recommendations varies among specialties, underscoring the need for further investigation into the real-world implementation of endocrine monitoring strategies for immunotherapy related endocrinopathies. One key challenge in clinical practice is the misdiagnosis of endocrine disorders due to symptom overlap with those arising from the underlying malignancy, concurrent cytotoxic treatments, or inappropriate screening methods. For instance, relying solely on TSH for screening may fail to detect central hypothyroidism, necessitating a comprehensive endocrine evaluation to distinguish primary from secondary causes [19]. Additionally, although rare, ICI-induced primary hypoparathyroidism can manifest as acute hypocalcemia, which, in severe cases, may be life-threatening if not promptly identified and managed [23]. With respect to calcium metabolism, routine calcium monitoring may be more clinically meaningful and pragmatic than routine PTH testing in patients receiving ICIs. When calcium abnormalities are detected, targeted evaluation with intact PTH, estimated GFR, and vitamin D levels is appropriate to guide further management and differential diagnosis. In our cohort, emergency hospitalizations due to preventable endocrine crises were observed, often resulting from late recognition of endocrine dysfunction and non-standardized surveillance practices, which occurred at a median of seven months after ICI initiation, typically around the 10th cycle of therapy. Given that ICI-induced endocrinopathies generally manifest within 3–6 weeks but can arise at any point during or after treatment, early and systematic monitoring is essential to ensure timely diagnosis and intervention [5, 24].

Although several guidelines provide structured recommendations for the management of irAEs, substantial inconsistencies persist regarding the timing and extent of endocrine surveillance [14, 15, 17, 25, 26]. Most primarily focus on baseline assessments and the management of clinically apparent irAEs, while guidance on continuous endocrine monitoring during and after ICI therapy remains insufficient. Oncology and endocrinology guidelines differ in their approaches to monitoring ICI-induced endocrinopathies. ASCO and ESMO emphasize structured thyroid and adrenal function monitoring, with ASCO recommending routine morning cortisol testing and ESMO favoring symptom-driven adrenal assessments [16, 17]. In contrast, endocrinology societies (ESE, French Endocrine Society (FES), British Society of Endocrinology (BSE)) advocate for a more comprehensive hormonal evaluation, particularly for patients at risk of hypophysitis. FES provides the most detailed recommendations for gonadal hormone screening, while BSE lacks clear guidance on ongoing endocrine monitoring [15, 24, 26]. Cost-effectiveness considerations should guide the extent of pituitary hormone testing and pretreatment endocrinology referral. A minimum standardized surveillance set targeting high-risk and clinically actionable axes may be more feasible than universal comprehensive panels in routine practice.

Our study highlights significant discrepancies between guideline recommendations and real-world clinical practice in the monitoring and management of endocrine irAEs. Despite the strong recommendations from ASCO, ESMO, and endocrine societies (ESE, FES, BSE) for early endocrine screening and routine follow-up, our findings reveal a suboptimal adherence to these guidelines in clinical practice [15, 16, 24, 26]. In our cohort, 21.5% of patients had no endocrine biochemical evaluation before ICI initiation, and only one patient underwent comprehensive endocrine assessment, despite ESE, FES, and BSE recommending a structured baseline evaluation. Thyroid function tests (TSH, fT4) were the most frequently assessed markers (78.4%), aligning with the recommendations of ASCO, ESMO, and ESE [15–17]. However, baseline morning cortisol, ACTH, and glucose levels were inconsistently measured, despite their inclusion in the French and British Endocrine Society guidelines [24, 26]. Gonadal function was rarely assessed (2.4%), even though FES and BSE recommend baseline LH, FSH, and testosterone in males and estradiol/FSH in females [24, 26]. These findings indicate that baseline endocrine screening was incomplete in most patients, which may have contributed to suboptimal recognition and management of endocrine irAEs in our study.

The low frequency of routine endocrine monitoring during ICI therapy in our cohort contrasts with ASCO and ESMO guidelines, which recommend repeat endocrine testing every 4–6 weeks [16, 17]. In our study, only 51.9% of patients underwent glycemic assessment, despite ESMO and FES emphasizing the importance of glucose monitoring, particularly for PD-1/PD-L1 inhibitors [17, 26]. Adrenal axis evaluation was notably inadequate, with only 25% of patients undergoing HPA axis assessment, and a complete adrenal workup performed in only 2 patients. This is in contrast with ASCO, ESE, and FES guidelines, which recommend routine cortisol monitoring, particularly for CTLA-4 inhibitors [15, 16, 26]. Pituitary MRI was performed in only 7 cases (6.9%), despite ESE and ASCO advocating for imaging when hypophysitis is suspected [15, 16]. These findings suggest that clinically significant adrenal dysfunctions may have been underdiagnosed, which aligns with the high rate of emergency department admissions due to hypocortisolism (76.9%) in our study. Furthermore, our data indicated that 93.5% of patients were referred to endocrinology only after the onset of an endocrine irAE, with proactive screening being rare (1.9%). Endocrinology consultations occurred at a median of 7.4 months after ICI initiation, often after completion of the 10th treatment cycle. This pattern likely reflects time-to-specialist involvement in routine practice rather than a definitive “referral delay” metric, given the heterogeneous timing of endocrine irAEs.

Thyroiditis (62.7%) and isolated ACTH deficiency (22.5%) were the most common endocrinopathies, consistent with the established risk profile of PD-1 inhibitors. However, the high incidence of secondary adrenal insufficiency in our cohort underscores the need for stricter adherence to adrenal function screening protocols. The high rate of emergency endocrinopathy-related admissions (16.7%), particularly due to adrenal crisis and thyrotoxicosis, highlights the consequences of inconsistent monitoring and delayed clinical recognition. While ASCO and ESMO recommend regular endocrine follow-up, our study suggests that these guidelines are not consistently implemented in clinical practice, leading to underdiagnosed endocrinopathies until symptomatic presentation [16, 17, 26]. Endocrinology guidelines (ESE, FES, BSE) provide more comprehensive recommendations for endocrine screening, yet our findings indicate limited adherence, particularly in adrenal and gonadal function monitoring [15, 24, 26]. The variations in axis assessment highlight the need for a more standardized approach in ICI-related endocrine evaluation.

## Limitations

This study has some limitations; Firstly this was a single-center study of patients referred to endocrinology, which may limit generalizability to all ICI-treated populations. The overall sample size was modest and some subgroups were sparse, contributing to wider confidence intervals. Given the retrospective design, some degree of missing data or misclassification is possible, and our primary comparisons were unadjusted, so residual confounding cannot be fully excluded. In addition, due to the retrospective nature of the study, we could not uniformly determine the exact timepoint of the first biochemical or radiographic evidence of endocrine toxicity across all patients. Therefore, the interval from ICI initiation to endocrinology consultation should be interpreted as time-to-specialist involvement rather than a definitive measure of referral delay. Future prospective studies with standardized surveillance protocols are warranted to quantify the lag between first abnormal findings and endocrinology referral.

## Conclusion

Endocrine irAEs are frequent and clinically significant toxicities of ICIs, yet their early recognition and biochemical surveillance remain heterogeneous in real-world practice. In our cohort, endocrinology involvement occurred largely after the onset of endocrine dysfunction and should be interpreted as time-to-specialist involvement rather than a definitive measure of referral delay, given the variable onset of endocrine toxicities across different axes. Nearly one in six patients presented with an endocrine emergency, spanning acute adrenal insufficiency and thyrotoxicosis to severe dysglycemia, demonstrating that endocrine irAEs can rapidly evolve into urgent and potentially life-threatening conditions if not anticipated.

By providing real-world data on monitoring practices, timing of endocrinology involvement, and emergency presentations, our findings highlight actionable gaps in routine care and reinforce the need for standardized surveillance pathways. Despite clear international guidance, more than one-fifth of patients underwent no baseline endocrine assessment, and comprehensive hormonal evaluation was exceedingly rare. The adrenal and gonadal axes, though clinically crucial, were among the least investigated.

A transition from reactive to proactive management is therefore essential. Systematic endocrine screening both before and throughout immunotherapy should become routine and be supported by multidisciplinary collaboration between oncologists and endocrinologists. Establishing standardized surveillance pathways may enable earlier detection, timely hormone replacement, and prevention of avoidable endocrine crises, ultimately improving both treatment safety and continuity of immunotherapy.

## Data Availability

No datasets were generated or analysed during the current study.
